# Crystal structure of bis­(acetato-κ*O*)bis­(pyridine-2-carboxamide oxime-κ^2^ *N*,*N*′)cadmium ethanol disolvate

**DOI:** 10.1107/S1600536814017978

**Published:** 2014-08-23

**Authors:** Jiyong Liu

**Affiliations:** a Department of Chemistry, Zhejiang University, Hangzhou, Zhejiang 310027, People’s Republic of China

**Keywords:** crystal structure, Cd^II^ complex, acetate, pyridine-2-carboxamide oxime, N—H⋯O hydrogen bonding

## Abstract

In the title compound, [Cd(CH_3_COO)_2_(C_6_H_7_N_3_O)_2_]·2C_2_H_5_OH, the Cd^II^ atom, which lies on a twofold rotation axis, is coordinated by two monodentate acetate groups and two *N*,*N*′-chelating pyridine-2-carboxamide oxime ligands, leading to a distorted octahedral coordination sphere. The mononuclear complex mol­ecules are assembled into chains along the *c*-axis direction *via* N—H⋯O hydrogen-bonding inter­actions. These chains are further assembled by O—H⋯O hydrogen bonds involving the ethanol solvent mol­ecules into a three-dimensional supramolecular structure.

## Abstract 2

In this Cd^II^ complex incorporating two monodentate acetate groups and two *N*,*N*′-chelating pyridine-2-carboxamide oxime ligands, mol­ecules are assembled into chains along the *c* axis *via* N—H⋯O hydrogen bonding. The resulting chains are further assembled by ethanol solvent mol­ecules into a three-dimensional supermolecular structure.

## Chemical context   

The monoanions of simple of 2-pyridyl oximes, (py)C(*R*)NOH (*R* = a non-coordinating group, *e.g.* H, Me, Ph *etc*.), are remarkable sources of homo- and heterometallic complexes with novel structures and inter­esting physical properties (Miyasaka *et al.*, 2003 ▶; Stamatatos *et al.*, 2007 ▶). A logical extension of such studies is the investigation of the coordin­ation chemistry of analogous organic mol­ecules in which the non-donor *R* group is replaced by a donor group such as pyridine, cyano *etc.* (Alcazar *et al.*, 2013 ▶; Escuer *et al.*, 2011 ▶). When *R* is an amino group, the resulting ligand is pyridine-2-amidoxime, (py)C(NH_2_)NOH, which belongs to the class of amidoximes. The presence of the amine functionality is expected to alter the coordination behaviour of this ligand in comparison with that of the (py)C(*R*)NOH (*R* = a non-coordinating group) ligands. The characteristics that differentiate the amino group are its coordination capability, potential for deprotonation, different electronic properties and hydrogen-bonding effects. 
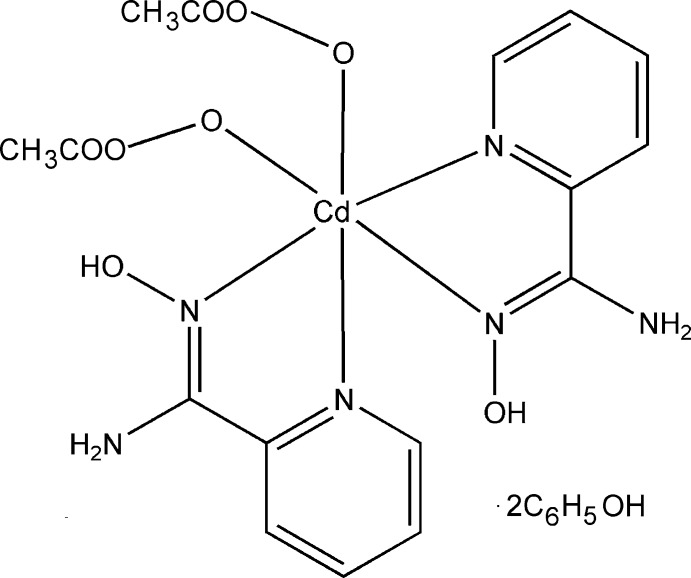


The present work reports the first use of (py)C(NH_2_)NOH in Cd^II^ coordination chemistry and describes the synthesis and structure of the mononuclear title compound.

## Structural commentary   

**Figure 1:**
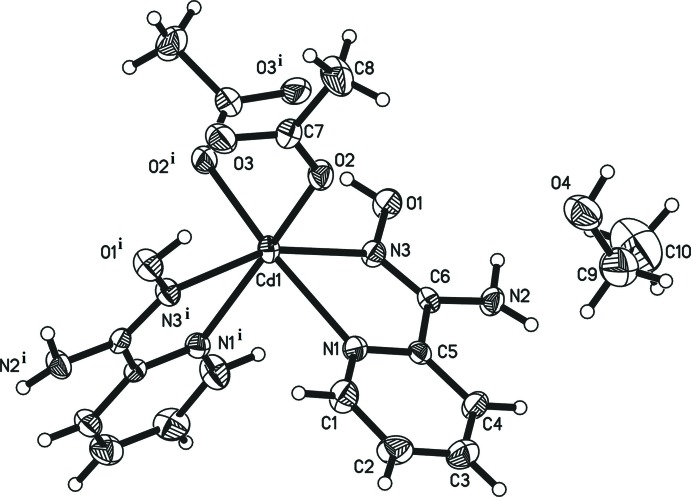
The title compound with displacement ellipsoids are drawn at the 30% probability level. [Symmetry code: (i) −*x* + 1, *y*, −*z* + 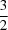.]

The title complex consists of isolated [Cd(O_2_CMe)_2_{(py)C(NH_2_)NOH}_2_] complex mol­ecules and ethanol solvent mol­ecules. The central Cd^II^ atom is located on a twofold rotation axis (Wyckoff site 4*e*). The Cd^II^ atom is coordinated by two monodentate MeCO_2_^−^ groups and two *N*,*N*′-chelating (py)C(NH_2_)NOH ligands (Fig. 1 ▶ and Table 1 ▶). The (py)C(NH_2_)NOH donor atoms are the N atoms of the neutral oxime and the 2-pyridyl groups. The amino N atom of each ligand remains uncoord­in­ating, albeit participating in an extensive inter­molecular hydrogen-bonding network. Each of the two coordinating (py)C(NH_2_)NOH mol­ecules results in the formation of a five-membered chelate ring including a Cd^II^ atom, in which the chelate angle N1—Cd1—N1 [86.7 (2)°] is noteably larger than comparable angles found in [Cd(HCO_2_)_2_(pya)_2_] (pya = pyridine-2-aldoxime**;** Croitor *et al.*, 2013 ▶).

## Supra­molecular features   

**Figure 2:**
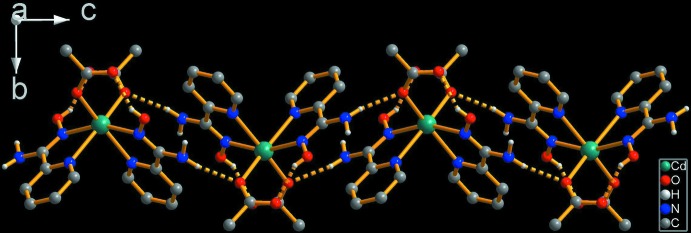
The hydrogen-bonded chain along the *c* axis. Dashed lines represent hydrogen bonds and H atoms bonded to C atoms have been omitted for clarity.

**Figure 3:**
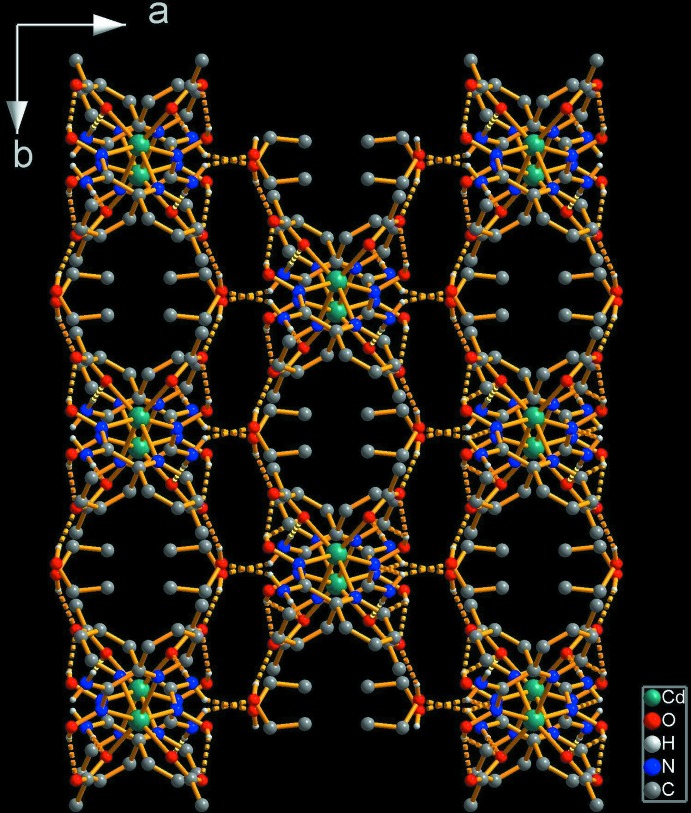
The crystal structure projected along the *c* axis. Dashed lines represent hydrogen bonds and H atoms bonded to C atoms have been omitted for clarity.

Table 2 ▶ shows the hydrogen-bonding inter­actions. There are two strong symmetry-related intra­molecular hydrogen bonds between the unbound oxime (–O1—H1) group and uncoordinating acetate atom O3. Uncoordinating amino atom N2 acts as a donor for two hydrogen bonds; in one of these, the acceptor is coordinating atom O2 from the acetate group, which leads to the formation of chains running along the *c-*axis direction (Fig. 2 ▶). These chains are further linked into a three-dimensional network by hydrogen bonds involving the ethanol solvent mol­ecule (O4), acting as a donor for the uncoord­in­ating carboxyl­ate O atom (O3) and as an acceptor for the remaining amino H atom H2*B* (Table 2 ▶ and Fig. 3 ▶).

## Synthesis and crystallization   

A stoichiometric amount of (py)C(NH_2_)NOH and Cd(OAc)_2_·3H_2_O in a 2:1 ratio was dissolved in 20 ml ethanol and 10 ml DMF, and the solution left to evaporate slowly to afford colourless block-like crystals after three weeks at room temperature.

## Refinement   

Crystal data, data collection and structure refinement details are summarized in Table 3 ▶. H atoms bonded to C atoms were placed in geometrically calculated position and were refined using a riding model, with C—H = 0.93 (aromatic) or 0.96 Å (methyl) and *U*_iso_(H) = 1.2*U*_eq_(C_aromatic_) and 1.5*U*_eq_(C_methyl_). The N- and O-bound H atoms were located in a difference map and the coordinates were refined with N—H = 0.86 (1) Å and *U*_iso_(H) = 1.2*U*_eq_(N) or 1.5*U*_eq_(O).

## Supplementary Material

Crystal structure: contains datablock(s) global, I. DOI: 10.1107/S1600536814017978/bg2533sup1.cif

Structure factors: contains datablock(s) I. DOI: 10.1107/S1600536814017978/bg2533Isup2.hkl

CCDC reference: 1017896

Additional supporting information:  crystallographic information; 3D view; checkCIF report

## Figures and Tables

**Table 1:** Selected bond lengths (Å)

Cd1—O2	2.288 (3)	Cd1—N3	2.315 (3)
Cd1—N1	2.413 (4)		

**Table 2:** Hydrogen-bond geometry (Å, °)

*D*—H⋯*A*	*D*—H	H⋯*A*	*D*⋯*A*	*D*—H⋯*A*
O1—H1⋯O3^i^	0.85 (1)	1.86 (4)	2.600 (5)	145 (6)
N2—H2*A*⋯O2^ii^	0.85 (1)	2.20 (2)	3.040 (5)	169 (5)
N2—H2*B*⋯O4	0.85 (1)	2.45 (4)	3.113 (6)	136 (5)
O4—H4*A*⋯O3^iii^	0.85 (1)	2.09 (3)	2.903 (5)	161 (8)

Symmetry codes: (i) 
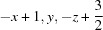
; (ii) 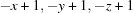; (iii) 
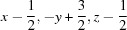
.

**Table 3:** Experimental details

Crystal data	
Chemical formula	[Cd(C_2_H_3_O_2_)_2_(C_6_H_7_N_3_O)_2_]·2C_2_H_6_O
*M*_r_	596.92
Crystal system, space group	Monoclinic, *C*2/*c*
Temperature (K)	294
*a*, *b*, *c* (Å)	15.894 (3), 10.9654 (17), 15.0212 (16)
β (°)	91.746 (12)
*V* (Å^3^)	2616.7 (7)
*Z*	4
Radiation type	Mo *K*α
μ (mm^−1^)	0.89
Crystal size (mm)	0.28 × 0.26 × 0.2

Data collection	
Diffractometer	Agilent Xcalibur, Atlas, Gemini ultra
Absorption correction	Multi-scan (*CrysAlis PRO*; Agilent, 2011 ▶)
*T*_min_, *T*_max_	0.910, 1.000
No. of measured, independent and observed [*I* > 2σ(*I*)] reflections	5661, 2400, 2017
*R*_int_	0.050
(sin θ/λ)_max_ (Å^−1^)	0.602

Refinement	
*R*[*F* ^2^ > 2σ(*F* ^2^)], *wR*(*F* ^2^), *S*	0.047, 0.125, 1.05
No. of reflections	2400
No. of parameters	173
No. of restraints	4
H-atom treatment	H atoms treated by a mixture of independent and constrained refinement
Δρ_max_, Δρ_min_ (e Å^−3^)	0.85, −0.48

Computer programs: *CrysAlis PRO* (Agilent, 2011 ▶), *SHELXS97* and *SHELXL97* (Sheldrick, 2008 ▶) and *OLEX2* (Dolomanov *et al.*, 2009 ▶).

## supplementary crystallographic information

### Crystal data

**Table:**

[Cd(C_2_H_3_O_2_)_2_(C_6_H_7_N_3_O)_2_]·2C_2_H_6_O	*F*(000) = 1224
*M**_r_* = 596.92	*D*_x_ = 1.515 Mg m^−^^3^
Monoclinic, *C*2/*c*	Mo *K*α radiation, λ = 0.71073 Å
*a* = 15.894 (3) Å	Cell parameters from 1816 reflections
*b* = 10.9654 (17) Å	θ = 2.9–29.6°
*c* = 15.0212 (16) Å	µ = 0.89 mm^−^^1^
β = 91.746 (12)°	*T* = 294 K
*V* = 2616.7 (7) Å^3^	Block, colourless
*Z* = 4	0.28 × 0.26 × 0.2 mm

### Data collection

**Table:**

Agilent Xcalibur, Atlas, Gemini ultra diffractometer	2400 independent reflections
Radiation source: Enhance (Mo) X-ray Source	2017 reflections with *I* > 2σ(*I*)
Graphite monochromator	*R*_int_ = 0.050
Detector resolution: 10.3592 pixels mm^-1^	θ_max_ = 25.4°, θ_min_ = 3.5°
ω scans	*h* = −19→16
Absorption correction: multi-scan (*CrysAlis PRO*; Agilent, 2011)	*k* = −13→11
*T*_min_ = 0.910, *T*_max_ = 1.000	*l* = −18→16
5661 measured reflections	

### Refinement

**Table:**

Refinement on *F*^2^	Primary atom site location: structure-invariant direct methods
Least-squares matrix: full	Secondary atom site location: difference Fourier map
*R*[*F*^2^ > 2σ(*F*^2^)] = 0.047	Hydrogen site location: inferred from neighbouring sites
*wR*(*F*^2^) = 0.125	H atoms treated by a mixture of independent and constrained refinement
*S* = 1.05	*w* = 1/[σ^2^(*F*_o_^2^) + (0.0689*P*)^2^] where *P* = (*F*_o_^2^ + 2*F*_c_^2^)/3
2400 reflections	(Δ/σ)_max_ < 0.001
173 parameters	Δρ_max_ = 0.85 e Å^−^^3^
4 restraints	Δρ_min_ = −0.48 e Å^−^^3^

### Special details

**Table:**

Geometry. All e.s.d.'s (except the e.s.d. in the dihedral angle between two l.s. planes) are estimated using the full covariance matrix. The cell e.s.d.'s are taken into account individually in the estimation of e.s.d.'s in distances, angles and torsion angles; correlations between e.s.d.'s in cell parameters are only used when they are defined by crystal symmetry. An approximate (isotropic) treatment of cell e.s.d.'s is used for estimating e.s.d.'s involving l.s. planes.
Refinement. Refinement of *F*^2^ against ALL reflections. The weighted *R*-factor *wR* and goodness of fit *S* are based on *F*^2^, conventional *R*-factors *R* are based on *F*, with *F* set to zero for negative *F*^2^. The threshold expression of *F*^2^ > σ(*F*^2^) is used only for calculating *R*-factors(gt) *etc*. and is not relevant to the choice of reflections for refinement. *R*-factors based on *F*^2^ are statistically about twice as large as those based on *F*, and *R*- factors based on ALL data will be even larger.

### Fractional atomic coordinates and isotropic or equivalent isotropic displacement parameters (Å^2^)

**Table:**

	*x*	*y*	*z*	*U*_iso_*/*U*_eq_	
Cd1	0.5000	0.55546 (4)	0.7500	0.0336 (2)	
O1	0.3289 (2)	0.5799 (3)	0.6185 (2)	0.0456 (9)	
H1	0.323 (4)	0.623 (5)	0.664 (2)	0.068*	
O2	0.5829 (2)	0.6898 (3)	0.67588 (19)	0.0448 (8)	
O3	0.6590 (3)	0.7760 (3)	0.7846 (2)	0.0627 (11)	
N1	0.5479 (2)	0.3954 (4)	0.6536 (2)	0.0349 (9)	
N2	0.3668 (3)	0.4189 (4)	0.5009 (3)	0.0411 (10)	
H2A	0.384 (3)	0.381 (4)	0.456 (2)	0.049*	
H2B	0.333 (3)	0.478 (3)	0.493 (4)	0.049*	
N3	0.4048 (2)	0.5170 (4)	0.6337 (2)	0.0348 (9)	
C1	0.6196 (3)	0.3347 (5)	0.6661 (3)	0.0510 (13)	
H1A	0.6528	0.3520	0.7166	0.061*	
C2	0.6469 (4)	0.2478 (5)	0.6083 (4)	0.0574 (14)	
H2	0.6969	0.2059	0.6200	0.069*	
C3	0.5988 (3)	0.2237 (5)	0.5326 (4)	0.0548 (14)	
H3	0.6165	0.1666	0.4914	0.066*	
C4	0.5238 (3)	0.2855 (4)	0.5189 (3)	0.0421 (12)	
H4	0.4900	0.2698	0.4686	0.051*	
C5	0.4994 (3)	0.3717 (4)	0.5812 (3)	0.0304 (10)	
C6	0.4200 (3)	0.4393 (4)	0.5716 (3)	0.0307 (10)	
C7	0.6314 (3)	0.7709 (5)	0.7064 (3)	0.0423 (12)	
C8	0.6594 (5)	0.8661 (6)	0.6421 (4)	0.077 (2)	
H8A	0.6782	0.8273	0.5890	0.115*	
H8B	0.7047	0.9125	0.6688	0.115*	
H8C	0.6132	0.9194	0.6273	0.115*	
O4	0.2093 (3)	0.5194 (5)	0.3968 (3)	0.0762 (13)	
H4A	0.207 (6)	0.583 (5)	0.364 (5)	0.114*	
C9	0.1674 (7)	0.4180 (8)	0.3623 (6)	0.105 (3)	
H9A	0.1766	0.3505	0.4032	0.126*	
H9B	0.1927	0.3961	0.3066	0.126*	
C10	0.0799 (7)	0.4315 (9)	0.3466 (8)	0.146 (5)	
H10A	0.0541	0.4565	0.4006	0.219*	
H10B	0.0563	0.3551	0.3271	0.219*	
H10C	0.0698	0.4922	0.3014	0.219*	

### Atomic displacement parameters (Å^2^)

**Table:**

	*U*^11^	*U*^22^	*U*^33^	*U*^12^	*U*^13^	*U*^23^
Cd1	0.0343 (3)	0.0403 (3)	0.0260 (3)	0.000	−0.00478 (18)	0.000
O1	0.0355 (19)	0.053 (2)	0.047 (2)	0.0123 (16)	−0.0101 (15)	−0.0073 (16)
O2	0.054 (2)	0.046 (2)	0.0348 (17)	−0.0145 (18)	−0.0026 (14)	−0.0009 (15)
O3	0.089 (3)	0.042 (2)	0.055 (2)	−0.020 (2)	−0.027 (2)	0.0086 (17)
N1	0.036 (2)	0.034 (2)	0.0340 (19)	0.0011 (18)	−0.0032 (16)	−0.0007 (16)
N2	0.043 (2)	0.045 (3)	0.034 (2)	0.005 (2)	−0.0100 (18)	−0.0086 (18)
N3	0.031 (2)	0.040 (2)	0.033 (2)	0.0060 (18)	−0.0039 (15)	−0.0039 (17)
C1	0.039 (3)	0.065 (4)	0.049 (3)	0.013 (3)	−0.012 (2)	−0.008 (3)
C2	0.048 (3)	0.051 (3)	0.073 (4)	0.019 (3)	−0.004 (3)	−0.002 (3)
C3	0.058 (3)	0.048 (3)	0.059 (3)	0.008 (3)	0.008 (3)	−0.009 (3)
C4	0.046 (3)	0.041 (3)	0.039 (2)	0.004 (2)	−0.001 (2)	−0.010 (2)
C5	0.035 (2)	0.028 (2)	0.028 (2)	−0.003 (2)	0.0008 (18)	0.0048 (17)
C6	0.031 (2)	0.035 (2)	0.026 (2)	−0.003 (2)	0.0000 (17)	0.0072 (18)
C7	0.043 (3)	0.046 (3)	0.038 (3)	−0.001 (2)	−0.001 (2)	−0.006 (2)
C8	0.097 (5)	0.075 (5)	0.057 (4)	−0.045 (4)	−0.008 (3)	0.020 (3)
O4	0.081 (3)	0.068 (3)	0.079 (3)	−0.001 (3)	−0.021 (2)	0.015 (2)
C9	0.148 (9)	0.080 (6)	0.086 (6)	−0.003 (6)	−0.029 (6)	0.001 (4)
C10	0.139 (10)	0.150 (11)	0.147 (9)	−0.070 (8)	−0.041 (8)	0.044 (7)

### Geometric parameters (Å, º)

**Table:**

Cd1—O2	2.288 (3)	C2—C3	1.376 (8)
Cd1—O2^i^	2.288 (3)	C3—H3	0.9300
Cd1—N1	2.413 (4)	C3—C4	1.381 (7)
Cd1—N1^i^	2.413 (4)	C4—H4	0.9300
Cd1—N3^i^	2.315 (3)	C4—C5	1.394 (6)
Cd1—N3	2.315 (3)	C5—C6	1.468 (6)
O1—H1	0.846 (10)	C7—C8	1.498 (7)
O1—N3	1.404 (5)	C8—H8A	0.9600
O2—C7	1.254 (6)	C8—H8B	0.9600
O3—C7	1.244 (5)	C8—H8C	0.9600
N1—C1	1.328 (6)	O4—H4A	0.851 (10)
N1—C5	1.339 (5)	O4—C9	1.388 (10)
N2—H2A	0.851 (10)	C9—H9A	0.9700
N2—H2B	0.847 (10)	C9—H9B	0.9700
N2—C6	1.355 (6)	C9—C10	1.412 (14)
N3—C6	1.291 (6)	C10—H10A	0.9600
C1—H1A	0.9300	C10—H10B	0.9600
C1—C2	1.368 (7)	C10—H10C	0.9600
C2—H2	0.9300		
			
O2—Cd1—O2^i^	99.86 (18)	C2—C3—C4	118.9 (5)
O2—Cd1—N1	88.81 (13)	C4—C3—H3	120.5
O2^i^—Cd1—N1	163.16 (12)	C3—C4—H4	120.4
O2—Cd1—N1^i^	163.16 (12)	C3—C4—C5	119.3 (4)
O2^i^—Cd1—N1^i^	88.81 (13)	C5—C4—H4	120.4
O2—Cd1—N3^i^	96.42 (12)	N1—C5—C4	120.8 (4)
O2—Cd1—N3	97.07 (12)	N1—C5—C6	117.0 (4)
O2^i^—Cd1—N3	96.42 (12)	C4—C5—C6	122.2 (4)
O2^i^—Cd1—N3^i^	97.07 (12)	N2—C6—C5	120.5 (4)
N1^i^—Cd1—N1	86.69 (19)	N3—C6—N2	123.3 (4)
N3—Cd1—N1	68.01 (13)	N3—C6—C5	116.1 (4)
N3—Cd1—N1^i^	96.27 (13)	O2—C7—C8	116.7 (4)
N3^i^—Cd1—N1^i^	68.01 (13)	O3—C7—O2	124.9 (5)
N3^i^—Cd1—N1	96.27 (13)	O3—C7—C8	118.3 (5)
N3^i^—Cd1—N3	159.0 (2)	C7—C8—H8A	109.5
N3—O1—H1	106 (4)	C7—C8—H8B	109.5
C7—O2—Cd1	129.4 (3)	C7—C8—H8C	109.5
C1—N1—Cd1	124.4 (3)	H8A—C8—H8B	109.5
C1—N1—C5	119.2 (4)	H8A—C8—H8C	109.5
C5—N1—Cd1	116.4 (3)	H8B—C8—H8C	109.5
H2A—N2—H2B	119 (5)	C9—O4—H4A	116 (6)
C6—N2—H2A	120 (4)	O4—C9—H9A	108.3
C6—N2—H2B	111 (4)	O4—C9—H9B	108.3
O1—N3—Cd1	124.9 (3)	O4—C9—C10	115.9 (9)
C6—N3—Cd1	122.3 (3)	H9A—C9—H9B	107.4
C6—N3—O1	112.6 (3)	C10—C9—H9A	108.3
N1—C1—H1A	118.5	C10—C9—H9B	108.3
N1—C1—C2	123.1 (4)	C9—C10—H10A	109.5
C2—C1—H1A	118.5	C9—C10—H10B	109.5
C1—C2—H2	120.7	C9—C10—H10C	109.5
C1—C2—C3	118.7 (5)	H10A—C10—H10B	109.5
C3—C2—H2	120.7	H10A—C10—H10C	109.5
C2—C3—H3	120.5	H10B—C10—H10C	109.5
			
Cd1—O2—C7—O3	17.9 (8)	N1—Cd1—N3—O1	−178.6 (4)
Cd1—O2—C7—C8	−164.0 (4)	N1—Cd1—N3—C6	−3.7 (3)
Cd1—N1—C1—C2	−177.2 (4)	N1^i^—Cd1—N3—C6	−87.6 (4)
Cd1—N1—C5—C4	176.5 (3)	N1—C1—C2—C3	1.3 (9)
Cd1—N1—C5—C6	−4.1 (5)	N1—C5—C6—N2	−178.9 (4)
Cd1—N3—C6—N2	−177.2 (3)	N1—C5—C6—N3	0.9 (6)
Cd1—N3—C6—C5	3.0 (5)	N3—Cd1—O2—C7	157.2 (4)
O1—N3—C6—N2	−1.7 (6)	N3^i^—Cd1—O2—C7	−38.9 (4)
O1—N3—C6—C5	178.5 (3)	N3^i^—Cd1—N1—C1	−13.3 (4)
O2^i^—Cd1—O2—C7	59.4 (4)	N3—Cd1—N1—C1	−178.9 (4)
O2^i^—Cd1—N1—C1	−155.5 (4)	N3^i^—Cd1—N1—C5	169.5 (3)
O2—Cd1—N1—C1	83.0 (4)	N3—Cd1—N1—C5	3.9 (3)
O2—Cd1—N1—C5	−94.2 (3)	N3^i^—Cd1—N3—O1	137.7 (3)
O2^i^—Cd1—N1—C5	27.3 (6)	N3^i^—Cd1—N3—C6	−47.4 (3)
O2^i^—Cd1—N3—O1	8.1 (4)	C1—N1—C5—C4	−0.8 (7)
O2—Cd1—N3—O1	−92.8 (3)	C1—N1—C5—C6	178.6 (4)
O2^i^—Cd1—N3—C6	−177.1 (4)	C1—C2—C3—C4	−1.7 (9)
O2—Cd1—N3—C6	82.1 (4)	C2—C3—C4—C5	0.9 (8)
N1—Cd1—O2—C7	−135.1 (4)	C3—C4—C5—N1	0.4 (7)
N1^i^—Cd1—O2—C7	−60.6 (6)	C3—C4—C5—C6	−179.0 (4)
N1^i^—Cd1—N1—C1	−80.8 (4)	C4—C5—C6—N2	0.5 (6)
N1^i^—Cd1—N1—C5	102.1 (3)	C4—C5—C6—N3	−179.7 (4)
N1^i^—Cd1—N3—O1	97.6 (3)	C5—N1—C1—C2	−0.1 (8)

Symmetry code: (i) −*x*+1, *y*, −*z*+3/2.

### Hydrogen-bond geometry (Å, º)

**Table:**

*D*—H···*A*	*D*—H	H···*A*	*D*···*A*	*D*—H···*A*
O1—H1···O3^i^	0.85 (1)	1.86 (4)	2.600 (5)	145 (6)
N2—H2*A*···O2^ii^	0.85 (1)	2.20 (2)	3.040 (5)	169 (5)
N2—H2*B*···O4	0.85 (1)	2.45 (4)	3.113 (6)	136 (5)
O4—H4*A*···O3^iii^	0.85 (1)	2.09 (3)	2.903 (5)	161 (8)

Symmetry codes: (i) −*x*+1, *y*, −*z*+3/2; (ii) −*x*+1, −*y*+1, −*z*+1; (iii) *x*−1/2, −*y*+3/2, *z*−1/2.
